# A role for glutathione in Parkinson’s disease modification

**DOI:** 10.4103/NRR.NRR-D-25-00375

**Published:** 2025-08-13

**Authors:** Jessica Keating, Ian Martin

**Affiliations:** Jungers Center for Neurosciences, Department of Neurology, Oregon Health and Science University, Portland, OR, USA; Department of Chemical Physiology and Biochemistry, Oregon Health and Science University, Portland, OR, USA

Oxidative stress has long been implicated as a driving force in neurodegenerative disease, with studies of human brain tissue and animal models revealing its important role. Parkinson’s disease (PD), in particular, highlights the selective vulnerability of neurons to the insults of reactive oxygen species. The motor symptoms of PD are caused by degeneration of dopamine neurons in the substantia nigra. These neurons experience increased oxidative stress due in part to highly active mitochondria that support their high bioenergetic demand and the generation of reactive oxygen species by dopamine metabolism (Watanabe et al., 2024). Glutathione, specifically in its reduced state (GSH), is a primary antioxidant whose deficiency has been implicated in PD and other neurodegenerative diseases (Bjørkland et al., 2021). The demonstrated neuroprotective effects of glutathione replenishment in animal PD models are at odds with less conclusive results in human clinical trials. This perspective piece outlines some of this preclinical and clinical evidence and proposes a way forward considering discrepancies between the human and animal data.

Human postmortem tissue provided early evidence that glutathione deficiency is associated with PD. Using high-performance liquid chromatography on tissue from disease-relevant brain areas, Sian et al. (1994) reported decreased GSH in PD brains. Their photometric enzyme activity analysis of glutathione synthesizing and metabolizing enzymes uncovered increased gamma-glutamyl transpeptidase, a glutathione degradative enzyme, in PD substantia nigra. As a degradative enzyme necessary for astrocytes to supply neurons with glutathione precursors, its increase in PD may represent compensation for a neuronal GSH deficit (Pérez-Sala et al., 2023). Our group’s recent work demonstrated a decrease in the catalytic subunit of the glutathione synthesis enzyme glutamate-cysteine ligase (*GCLC*) in the brains of PD patients with the LRRK2 G2019S mutation, consistent with decreased brain glutathione synthesis in PD (Coleman et al., 2024). Postmortem tissue studies are complemented by magnetic resonance spectroscopy confirming decreased glutathione in the substantia nigra of people with PD (Shukla et al., 2023). Evidence for a causative role of deficient glutathione synthesis and related oxidative stress in dopamine neurodegeneration comes from animal models. We have shown that Drosophila strains experiencing age-related dopamine neuron loss have less glutathione in their head tissue as well as lower expression of the *Drosophila GCLC* ortholog *gclc*, and that neuronal overexpression of *gclc* rescues the neurodegenerative phenotype (Coleman et al., 2024). Relatedly, neuron-specific *GCLC* knockout in mice leads to mitochondrial dysfunction and neurodegeneration (Hashimoto et al., 2023). This and other work not discussed here due to space limitations have situated glutathione deficiency and ensuing oxidative stress as drivers of age-related neurodegeneration.

In animal and cell PD models, increasing the level of glutathione either by overexpression of synthesis enzymes or supplementation of its precursor N-acetylcysteine (NAC) protects against PD-related pathology. For example, in our study of *Drosophila* strains susceptible to age-related dopamine neuron loss, we found that neuronal overexpression of *gclc* rescues dopaminergic neurodegeneration as well as these strains’ particular susceptibility to oxidative stress induced by H_2_O_2_ treatment (Coleman et al., 2024). Treatment with NAC confirmed to increase glutathione levels in the substantia nigra, rescues levels of the dopamine synthesis enzyme tyrosine hydroxylase in a rat model of PD (Virel et al., 2019). Similarly, induced pluripotent stem cell-derived dopamine neurons bear an alpha-synuclein mutation that causes PD to produce less dopamine, which is rescued by GSH replenishment (Stykel et al., 2025). Consistent evidence for depleted glutathione in human PD brains as well as the encouraging results from bolstering glutathione levels in PD models have situated glutathione as a target for disease-modifying therapies.

Glutathione and its precursor NAC have both been the subject of clinical trials enrolling PD patients. Evidence from mammalian models suggests that both can cross the blood-brain barrier and magnetic resonance spectroscopy in human studies similarly indicates that intravenous NAC and intranasal glutathione administration lead to an increase in brain glutathione (Holmay et al., 2013; Mischley et al., 2017). Intravenous NAC and intranasal glutathione are both safe and well tolerated, but their efficacy remains to be conclusively determined. Mischley et al. (2017) conducted a Phase II clinical trial of intranasal glutathione that showed an improvement in PD symptoms especially among patients receiving the higher of two doses tested, but interpretation of results was hindered by unexpected and statistically equal improvement in the placebo group. Monti et al. (2019) found a similarly encouraging but modest improvement in PD symptoms with the administration of intravenous plus oral NAC, although their results were difficult to interpret due to the absence of a placebo group and therefore no patient blinding or control for the effect of study participation. Their study included DaTscan to quantify dopamine transporter binding (i.e., reuptake of dopamine from the synaptic cleft) in the striatum, which correlated with disease severity measured using a widely used clinical PD scale. With DaTscan, the authors demonstrated increased dopamine transporter binding following NAC administration. Both clinical studies reported here, as well as the prior smaller studies in PD patients that motivated their work, have been underpowered to evaluate treatment efficacy and involved control group features that made it difficult for the authors to draw confident conclusions about their well-motivated interventions. Further, short-term trials in general can miss longer-term effects of an intervention, which may be particularly important in protracted disease processes such as PD.

Notably, the studies reported above lasted for a maximum of four months. While data from animal models encourage glutathione targeting for disease modification, the design of these relatively short-term trials is best suited to measure changes in PD symptoms as opposed to frank disease modification. Monti et al.’s (2019) results showing increased dopamine transporter binding following NAC administration may support this point of symptom treatment as opposed to disease modification. Considering that increased dopamine signaling is the mainstay of PD symptom management (i.e., administration of carbidopa/levodopa and other adjunctive therapies to increase dopamine in the synaptic cleft), it is possible that symptom reduction attributable to glutathione or NAC is not due to dopamine neuron rescue per se. Many of the proteins involved in neurotransmission and neuromodulation are sensitive to redox state, which in turn is governed at least partly by the antioxidant glutathione. Indeed, blocking glutathione synthesis attenuates the neuromodulatory effect of dopamine in postsynaptic neurons *in vitro* (Steullet et al., 2008). Since glutathione can influence dopamine signaling, we cannot rule out that any effects of its relatively short-term administration target a symptom rather than cause of PD.

Further, trials involving PD patients occur during a time in the disease process when neurodegeneration has already taken hold; once PD is clinically evident, 30% to 70% of neurons die in disease-affected brain regions (Cheng et al., 2010). While saving neurons that remain at diagnosis is a worthy goal, the benefits of glutathione in animal studies that model early pathology prior to frank neurodegeneration compel us to investigate earlier in the disease course. Future studies involving people diagnosed with rapid eye movement (REM) sleep behavior disorder would support this goal. REM sleep behavior disorder is a prodrome of PD, with a distinct clinical presentation despite similar pathological hallmarks and a lifetime risk of phenoconversion to PD of almost 100% (Postuma, 2022). Therefore, to access the time window for glutathione treatment shown to be effective in animal models of PD, trials enrolling REM sleep behavior disorder patients may better assess disease modification as opposed to solely symptom management.

Herein lies the challenge of neurodegenerative disease trials. To test the efficacy of a putative disease-modifying therapy, a trial must begin with a prodromal or very early disease population and involve years or even decades of continued patient involvement and data collection. Increasing the duration of a trial increases the likelihood of variability in the study sample as participants’ environments and physiology changes over time, requiring large cohorts for sufficient power. The authors who conducted the clinical studies and trials reported above have undertaken immense work to glean information about the role of glutathione in improving PD symptoms. Assessing the true potential of glutathione for disease modification in human patients demands an order of magnitude greater commitment of time, resources, and collaboration. Overcoming these challenges is essential if we are to move beyond symptom management and toward therapies that can alter the course of PD and other neurodegenerative diseases.

*This work was supported by OHSU Neurology Foundation Funds and NIH Grant R01NS119226 to IM.*
